# Prevalence of Congenital Heart Diseases in Dogs in Tehran, Iran: A Retrospective Study From 2013 to 2023

**DOI:** 10.1155/vmi/2994461

**Published:** 2025-02-18

**Authors:** Zeynab Pourghasemi, Nika Norouzi, Narges Safari, Hiva Khakpour, Donya Keypoori, Farzane Shams, Arman Abdous, Mohammad Jokar

**Affiliations:** ^1^College of Veterinary Medicine, Tabriz University, Tabriz, Iran; ^2^Faculty of Veterinary Medicine, Karaj Branch, Islamic Azad University, Karaj, Iran; ^3^College of Veterinary Medicine, Garmsar Branch, Islamic Azad University, Semnan, Iran; ^4^College of Veterinary Medicine, Shabestar Branch, Islamic Azad University, Tabriz, Iran; ^5^Faculty of Veterinary Medicine, Urmia Branch, Islamic Azad University, Urmia, Iran; ^6^Graduate School for Cellular and Biomedical Sciences, University of Bern, Bern, Switzerland; ^7^Faculty of Veterinary Medicine, University of Calgary, Calgary AB T2N 1N4, Canada

**Keywords:** canine health, echocardiography, epidemiology, genetic predisposition, veterinary cardiology

## Abstract

Congenital heart disease (CHD) is a major health issue in dogs, contributing to both morbidity and mortality. This retrospective study reviews the epidemiological features and prevalence of CHD in dogs visiting veterinary facilities in Tehran, Iran, over the last 10 years. Medical records were analyzed for 4033 canines that underwent comprehensive cardiac examinations, including echocardiography, between January 2013 and October 2023. In this study, 88 cases of CHD were detected, and an overall prevalence of 2.18% was determined. A significant difference was noted between mixed-breed dogs (8.65%) and purebred dogs (1.63%). Pulmonary stenosis (PS) is the most commonly diagnosed CHD, followed by subaortic stenosis (SAS) and patent ductus arteriosus (PDA). CHD prevalence correlated strongly with age and gender; in particular, females and older dogs were more likely to suffer from specific CHDs. CHD is most often diagnosed without symptoms, highlighting the importance of regular screenings and careful auscultation for early detection. Future research must focus on identifying the genetic factors that make dogs more susceptible to CHDs and developing more effective methods for diagnosing and treating these conditions in canine populations. This study does not represent the general dog population in the region or the country but provides researchers with valuable insights into the epidemiology of CHD in dogs referred to veterinary hospitals in Tehran, Iran, underlining the importance of monitoring and focused therapies to improve their health and general well-being.

## 1. Introduction

Dogs with congenital heart disease (CHD) have structural and functional abnormalities in their hearts or major blood vessels that appear during particular developmental phases of embryonic development and continue to exist following birth [1]. This congenital abnormality compromises the heart's ability to regulate arterial and venous pressures, as well as the supply of oxygenated blood to tissues [2]. The presence of CHD is therefore an important risk factor for illness and death in dogs who are less than 1 year of age [3]. The majority of congenital cardiovascular abnormalities cause death shortly after birth, but some congenital heart defects (CHDs) may not show any symptoms and go unrecognized until a later stage in life [4, 5]. It is important to detect the condition early and to perform surgical interventions in order to prolong the life of the affected dog as well as contribute to breeding efforts aimed at reducing the prevalence of CHD in the future [6].

Understanding the epidemiology of CHDs is essential in order to prevent the spread of these diseases and keep dogs healthy. Research on the genetic factors has been conducted extensively in humans. However, there is limited information on the genetic factors that contribute to CHD in animals. Consequently, it is difficult to determine the actual impact of heredity on canine CHD [7]. Retrospective studies can provide a better understanding of the relative risks of certain heart diseases across different breeds of dogs, which can prove to be of great value in this context [8].

Conducting an epidemiological study of canine CHD offers valuable insights into the prevalence and risk factors associated with this condition. Geographical variations and diverse dog populations provide a comprehensive view across different segments of the canine population. Furthermore, retrospective studies allow for the examination of a wide range of historical data, enabling researchers to track trends and identify long-term changes in the occurrence of CHD [3, 6]. This study aimed to determine the prevalence of congenital cardiac disease in dogs brought to veterinary facilities in Tehran, Iran, over a 10-year period. The study examined the relationships between CHD and factors such as gender, breed, and age.

## 2. Materials and Methods

A retrospective study was conducted on data collected over 10 years from five veterinary hospitals in Tehran, Iran. In this study, only private veterinary hospitals were included. Reviewers evaluated medical records from January 2013 to October 2023. These databases contain complete medical records, including the history of each patient, physical examination findings, complementary examination reports, and echocardiography data.

### 2.1. Study Population

The study included all dogs that presented to the cardiology departments at five veterinary hospitals, where **echocardiography** was used as the primary diagnostic tool to diagnose **CHD**. Demographic information such as sex, age, breed, medical history, and physical examination results was also recorded. In addition to echocardiography, other cardiac assessments were performed, but the study relied solely on echocardiographic findings for confirming the diagnosis. A single cardiologist observed the data collection process across all five veterinary centers. Initially, all 4033 dogs underwent a radiological evaluation, after which **echocardiography** was conducted to provide a comprehensive assessment. Cardiologists reviewed the medical records of each patient, confirming the diagnosis of CHD, and identifying conditions such as **pulmonary stenosis (PS), subaortic stenosis (SAS),** and **patent ductus arteriosus (PDA).**

### 2.2. Diagnostic Procedures

In this study, Doppler echocardiography was performed as the gold-standard method of diagnosing CHD in animals [9]. Only patients with diagnoses confirmed by this method were included in this study. A range of echocardiographic techniques was utilized, including **two-dimensional (2D)** imaging, **M-mode, spectral Doppler**, and **color Doppler**, to assess cardiac structure and blood flow, identify abnormalities, and determine the presence and severity of CHD.

### 2.3. CHDs

The following CHDs found in this study are atrial septal defect (ASD), mitral dysplasia (MD), PDA, persistent left cranial vena cava (PLCVC), pulmonic stenosis (PS), reversed PDA (RPDA), SAS, tetralogy of Fallot (TF), tricuspid dysplasia (TD), valvular aortic stenosis (VAS), and ventricular septal defect (VSD).

The diagnostic criteria for each CHD, including echocardiographic, radiographic, and electrocardiographic parameters, are detailed in the accompanying table (Table 1) [9–14].

### 2.4. Equipment and Techniques

Echocardiographic evaluations were performed by board-certified veterinary cardiologists in accordance with the guidelines provided by the American College of Veterinary Internal Medicine (ACVIM). All examinations were carried out using three different ultrasound systems: the **GE Vingmed Ultrasound AS Vivid 7** system with a phased-array transducer, **Tissue Doppler Imaging (TDI)** mode, the **Mindray Bio-Medical Electronics Co. M5 Ultrasound system** (China) with a 7 MHz microconvex probe for portable assessments, and the **SonoSite Titan Ultrasound system**(USA) equipped with a microconvex probe operating at frequencies between 5.5 and 8 MHz. These systems facilitated a thorough evaluation of cardiac structure and function, utilizing **Doppler** and **color Doppler** techniques to assess blood flow, measure velocities, and identify CHDs in the study population.

### 2.5. Data Analysis

Statistical analysis required the classification of dogs into either “puppies” (less than a year of age) or “adults” (more than a year of age). Furthermore, a comparison was made between purebred and mixed-breed dogs. Some of the clinical signs that can be used to classify dogs as symptomatic are weakness, exercise intolerance, coughing, syncope, ascites, tachypnea, dyspnea, and cyanosis. Asymptomatic dogs did not show any physical signs of cardiac illness or had symptoms that were unrelated to it. This descriptive statistical analysis consisted of computing percentages and identifying the overall prevalence of CHD, as well as its prevalence under specific circumstances. The prevalence of CHD in dogs was determined by dividing the number of canine cases by the total number of dogs examined. An odds ratio (OR) for CHD was calculated by Fisher's exact test, with a significance threshold of 0.05. An R program was used to conduct the statistical analysis [15].

## 3. Result

**Figure 1:**
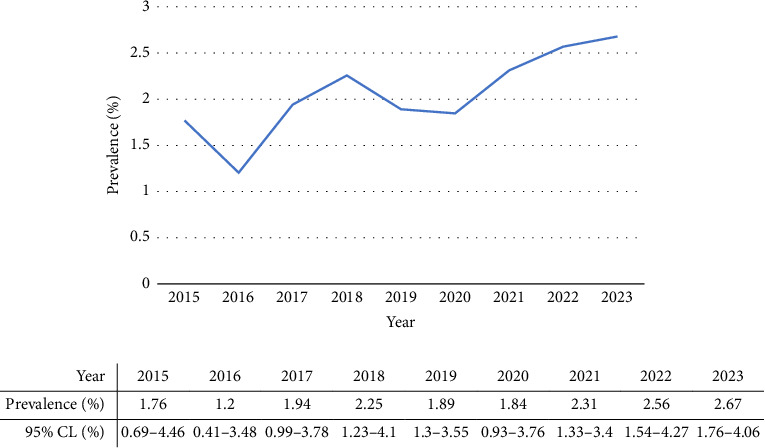
The trend of prevalence of congenital heart disease in dogs from 2015 to 2023.

Over 10 years, 4033 dogs were presented to the cardiology sections of five veterinary hospitals in Tehran, the capital city of Iran. A total of 88 dogs were diagnosed with CHDs, representing a prevalence of 2.18%. In Table 2, the prevalence of CHD in different breeds was shown, whereas, in Table 3, the prevalence of different congenital heart conditions was presented. Moreover, the prevalence and occurrence of CHDs were shown per year (Figure 1). Overall trends in CHD prevalence were increasing during the study period.

Among the 88 patients, 60% were female and 40% were male, resulting in a prevalence of CHD of 3.06% and 1.51%, respectively, among females and males. In addition, 80% of patients were adult dogs (> 1 year old), so the prevalence of CHD in this group (2.35%) was higher than that of puppies (1.66%, < 1 year old). This study identified 29 distinct breeds of dogs in addition to mixed breeds. There was a higher prevalence of CHD among mixed-breed dogs (8.65%) than among pure-breed dogs (1.63%). Dachshunds (5.71%), Maltese (5.59%), and Labradors (4.16%) ranked highest among purebred dogs (Table 2).

At the time of diagnosis, 72% of the patients were asymptomatic, while 28% were symptomatic, exhibiting signs such as coughing, exercise intolerance, difficulty breathing, fainting or collapse, fatigue or weakness, and cyanosis. According to cardiac auscultation, 35 dogs of patients had heart murmurs, but specifics regarding the type of murmurs were not available. Electrocardiographic tests were conducted on 48 dogs diagnosed with CHD, revealing various cardiac irregularities. Among these, 24% displayed either sinus bradycardia or sinus tachycardia, while 4% had atrial fibrillation. Additionally, 1% of the dogs showed an atrioventricular block of the first and second degree, and 2% exhibited a right bundle branch block. Isolated premature ventricular complexes were found in 5% of the cases. Other notable findings included 1% with ventricular tachycardia, 2% with ventricular fibrillation, and 3% with other arrhythmias. Furthermore, 6% showed signs of chamber enlargement, 2% had evidence of ventricular hypertrophy, and 3% exhibited evidence of atrial enlargement.

In 71 cases, thorax radiography was performed and revealed cardiomegaly (80% of cases), pulmonary congestion (70%), pleural effusion (50%), pulmonary edema (40%), caudal vena cava enlargement (30%), and pulmonary arterial enlargement (20%). Subjective evaluation, including the vertebral heart scale measurement, aids in diagnosis and assessment. A total of 11 types of CHDs were detected in the patients, of which PS (0.495%), SAS (0.421%), and PDA (0.222%) were the most prevalent.

In CHD dogs, PS was more prevalent in adults (31.25%) than in puppies (12.5%), and in mixed breed dogs (37.03%) than in pure breeds (16.39%). In other words, the adult group is approximately three times more likely (OR: 3.1818, 95% CL: 1.0399–9.7353) to have PS compared to the puppy group. Similarly, mixed-breed dogs are approximately three times more likely (OR: 3.001, 95% CL 1.0665–8.4388) to suffer from PS than pure-breed dogs (Table 4).

Additionally, SAS prevalence is higher among adults (29.16%) and female dogs (31.57%) than among puppies (7.5%) and male dogs (14%). The risk of SAS is approximately four times higher (OR: 4.2883, 95% CL: 1.1329–16.2316) in adult dogs and approximately more than twice as high (OR: 2.7937, 95% CL: 0.9795–7.9682) in female dogs. As the prevalence of PDA is higher in female dogs (18.42%) than in male dogs (4%), the risk of this CHD is about five times (OR: 5.4194, 95% CL: 1.0564–27.8012) higher in females. However, the prevalence of ASD is higher in male dogs (14%) than in female dogs (2.63%); therefore, male dogs are six times more likely to develop this disease than female dogs (OR: 6.8372, 95% CL: 0.806–57.9985) (Table 4).

## 4. Discussion

A 10-year study conducted in five veterinary hospitals in Tehran, Iran, reported a prevalence of 2.18% CHD in dogs. The prevalence rate of this disease is higher than the 1.6% reported in a Brazilian survey of 6710 dogs [8]. However, it falls within the broad range of 2.7%–23.5% observed in other studies across the globe [12, 13, 16, 17]. In contrast, it is much higher than the 0.13% reported in a shelter survey of 76,301 mixed-breed dogs [14]. There are several explanations for the differences in prevalence rates observed in different studies, including differences in study locations, demographics, and the proportion of mixed-breed and purebred dogs. The high prevalence of CHD (8.65%) in mixed-breed dogs in this study may be due to the significant number of mixed-breed dogs that visited veterinary hospitals. In comparison to the prevalence of each breed, mixed-breed dogs had a lower prevalence (0.95%). Therefore, demographic factors have a significant impact on the prevalence of CHD. In purebred dogs, Dachshunds (5.71%), Maltese (5.59%), and Labradors (4.16%) had the highest prevalence rates of CHD.

In this study, PDA, PS, and SAS were the most commonly diagnosed CHDs. Similar results have been obtained in other countries as well [8]. Although the prevalence of PDA in this study was 10.23%, it is lower than earlier investigations that documented higher rates [13, 16]. During the study period, the lower incidence of PDA may be attributed to a reduced number of cases that were recommended for surgical intervention in the study regions.

Based on the results of this study, it was found that the prevalence of ASD was higher than in previous studies at 9.09% [13, 16, 18, 19]. The disparity may be due to a lack of routine echocardiographic examinations and the increasing popularity of Doppler equipment for the detection of asymptomatic cases in older animals or animals with cardiac murmurs. In another study, it was found that advanced diagnostic techniques can be more effective in detecting ASDs [20].

The correlation between PS, adult dogs, and mixed breeds was an important discovery. The prevalence of PS was approximately three times higher in adult dogs compared to puppies, and mixed-breed dogs were also at a higher risk than purebred dogs. The prevalence of SAS was higher in females and adults, with females having twice as much SAS as males and adults having four times as much SAS as females. PDA was also significantly more prevalent in females, which is consistent with previous research [21].

The findings of this investigation also emphasize the importance of an early and accurate diagnosis of CHD. Seventy-two percent of the dogs did not show any symptoms at the time of their diagnosis, emphasizing the importance of regular checkups and careful listening for early identification. Approximately 40% of the patients had heart murmurs, which highlights the importance of comprehensive physical examinations. The electrocardiograms of 48 dogs revealed a variety of cardiac anomalies, with sinus bradycardia or tachycardia being the most frequent abnormalities found. A limited number of dogs suffered from more serious arrhythmias, including atrial fibrillation and ventricular tachycardia. Consequently, the results support the importance of electrocardiographic monitoring in dogs with CHD, which serves to identify and control potentially fatal arrhythmias. It is also important to perform a thoracic radiography in order to diagnose canines with CHD, as it often reveals abnormalities such as cardiomegaly, pulmonary congestion, and pleural effusions. In particular, echocardiographic examinations are critical for confirming diagnosis, particularly in asymptomatic dogs, which emphasized the need for accurate diagnostic methods.

In this study, the majority of dogs were classified as adults at the time of diagnosis based on their age distribution evaluation. On the other hand, some studies indicate that CHDs are more common in younger dogs [12, 14, 16, 18]. This study found a higher prevalence of the disease among adult dogs, perhaps because milder forms of the disease are sometimes not detected until later in life, or because more dogs are being examined in Tehran as diagnostic skills have improved over time. Despite the variations in prevalence and presentation of CHD across regions, the results of this study provide significant epidemiological information about dogs' condition in Tehran, Iran. It appears that certain purebred dogs, such as Dachshunds, Maltese, and Labradors, as well as mixed breed dogs, may be more prone to CHD. Additionally, some CHDs, such as SAS and PDA, appear to be affected by the gender and age of the dogs.

In most cases, controlling CHD requires long-term medication or surgical procedures that have a significant financial impact on the owner [2]. Early identification and treatment can greatly improve the quality of life and the lifespan of sick dogs. Research such as this emphasizes the need for regular health examinations, especially for breeds that have an increased risk of CHD. These examinations should include diagnostic imaging and cardiac auscultation.

It is important to consider several limitations of this study. Considering the retrospective nature of this study and the reliance on medical records, it is possible that some cases may have been underreported or misclassified. This study involved dogs that were presented at five veterinary hospitals in Tehran, which may not represent the general dog population in the region or in other parts of the country. In addition, the relatively small number of CHD cases limits the generalizability of the results.

## 5. Conclusion

The prevalence of CHD in this study was 2.18%, with PS, SAS, and PDA being the most common conditions diagnosed. There was a significant association between CHD prevalence and factors such as breed, age, and sex, which provides a research basis and improves diagnostic strategies in veterinary cardiology. Moreover, the findings emphasize the importance of routine screenings and advanced diagnostic tools in the early detection and management of CHD in dogs. This information will enhance veterinary care and guide future research by allowing a better understanding of the epidemiological characteristics of CHD in dogs in Tehran.

## Figures and Tables

**Table 1:** Diagnostic criteria for congenital heart diseases (CHDs) in dogs.

Congenital heart disease	Diagnostic criteria	Echocardiographic findings
Atrial septal defect (ASD)	Flow across the septum during diastole; shunt confirmation via contrast	Diastolic flow velocity of 1.0–1.5 m/s across septum; shunt confirmation
Mitral dysplasia (MD)	Abnormal mitral valve structure, regurgitation, left atrium enlargement	Mitral regurgitation; regurgitant jet velocity ≥ 2.5 m/s; LA > 1.5 cm
Patent ductus arteriosus (PDA)	Continuous ductal flow; left heart dilation	Continuous ductal flow > 2.5 m/s; left ventricle dilation > 2 cm
Persistent left cranial vena cava	Dilated coronary sinus; abnormal venous return	Coronary sinus diameter > 0.5 cm; abnormal venous flow
Pulmonic stenosis (PS)	Pulmonic valve malformation, narrowed outflow tract, right heart dilation	Right ventricular outflow velocity > 2.5 m/s; poststenotic dilation > 1.5 cm
Reversed patent ductus arteriosus	Right-to-left shunting; right heart dilation	Right-to-left shunt flow > 3 m/s; right ventricle dilation > 2 cm
Sub-aortic stenosis (SAS)	Subvalvular fibrous ridge; left ventricular hypertrophy	Aortic outflow velocity > 2.3 m/s; left ventricular hypertrophy > 2 cm
Tetralogy of fallot (TF)	Right ventricular outflow obstruction, VSD, overriding aorta	VSD size > 0.5 cm; right ventricular hypertrophy > 2 cm; overriding aorta
Tricuspid dysplasia (TD)	Abnormal tricuspid valve structure, right atrium enlargement, regurgitation	Tricuspid regurgitation jet velocity > 2.3 m/s; right atrium > 1.5 cm
Valvular aortic stenosis (VAS)	Flow across the valve during systole; shunt confirmation via contrast	Systolic flow velocity of 1.0–1.5 m/s across the valve; shunt confirmation
Ventricular septal defect (VSD)	Abnormal ventricular septum with flow from left to right ventricle	Left-to-right shunt with jet velocity > 2.5 m/s

**Table 2:** The prevalence of congenital heart disease (CHD) was diagnosed in 88 study dogs based on their breeds.

Breed	Total N	Cases	Prevalence of	
			CHD per breed %	Each breed within the CHD dogs %
Mixed	312	27	8.653	30.682
Dachshund	35	2	5.714	2.272
Maltese	143	8	5.594	9.09
Labrador	72	3	4.166	3.409
Golden retriever	173	6	3.468	6.818
Beagle	101	3	2.97	3.409
Bulldog	75	2	2.666	2.272
Shih Tzu	187	4	2.139	4.545
Pug	192	4	2.083	4.545
Boxer	97	2	2.061	2.272
Yorkshire Terrier	180	3	1.666	3.409
Basset Hound	60	1	1.666	1.136
Pomeranian	188	3	1.595	3.409
Great Dane	128	2	1.562	2.272
German shepherds	195	3	1.538	3.409
Chow Chow	154	2	1.298	2.272
Sarabi	84	1	1.19	1.136
Cocker Spaniel	170	2	1.176	2.272
Samoyed	175	2	1.142	2.272
Rottweiler	189	2	1.058	2.272
Dalmatian	96	1	1.041	1.136
Cane Corso	111	1	0.9	1.136
Akita	123	1	0.813	1.136
Dobermann	142	1	0.704	1.136
Chihuahua	154	1	0.649	1.136
Husky	203	1	0.492	1.136
Corgi	82	0	0	0
Shar-pei	60	0	0	0
Newfoundland	54	0	0	0
Tibetan Mastiff	98	0	0	0
Total	4033	88	2.181	100

**Table 3:** The prevalence of different types of congenital heart diseases was diagnosed in 88 study dogs.

Congenital heart disease	Number of cases	Prevalence of caseload	Prevalence of congenital heart disease
Pulmonic stenosis (PS)	20	0.495	22.727
Subaortic stenosis (SAS)	17	0.421	19.318
Patent ductus arteriosus (PDA)	9	0.223	10.227
Atrial septal defect (ASD)	8	0.198	9.09
Tricuspid dysplasia (TD)	8	0.198	9.09
Ventricular septal defect (VSD)	7	0.173	7.954
Mitral dysplasia (MD)	6	0.1487	6.818
Valvular aortic stenosis (VAS)	5	0.123	5.681
Tetralogy of Fallot (TF)	4	0.099	4.545
Persistent left cranial vena cava (PLCVC)	3	0.074	3.409
Reversed patent ductus arteriosus (RPDA)	1	0.024	1.136

**Table 4:** The risk factors reached a level of statistical significance (*p* < 0.05) in the 88 dogs based on the type of congenital heart disease (CHD).

	Related factor	Prevalence among CHD dogs %	*p*-value	Odds ratio	95% confidence intervals
PS	Adult	31.25	0.0316	3.1818	1.0399–9.7353
	Puppy	12.5			
	Pure	16.39	0.0341	3.001	1.0665–8.4388
	Mixed	37.03			

SAS	Adult	29.16	0.0203	4.2883	1.1329–16.2316
	Puppy	7.5			
	Female	31.57	0.0446	2.7937	0.9795–7.9682
	Male	14			

PDA	Female	18.42	0.0316	5.4194	1.0564–27.8012
	Male	4			

ASD	Female	2.63	0.0474	6.8372	0.806–57.9985
	Male	14			

Abbreviations: ASD, atrial septal defect; PDA, patent ductus arteriosus; PS, pulmonic stenosis; SAS, subaortic stenosis.

## Data Availability

The data that support the findings of this study are available from the corresponding author upon reasonable request. Due to privacy concerns, individual medical records cannot be shared publicly, but aggregate data are accessible upon request.
